# Die EU-Verordnung für In-vitro-Diagnostika (IVDR) in der Praxis: Umsetzung und Anwendung – Ergebnisse einer öffentlichen Veranstaltung der Arbeitsgemeinschaft der Wissenschaftlichen Medizinischen Fachgesellschaften (AWMF) im März 2023

**DOI:** 10.3205/000327

**Published:** 2024-01-29

**Authors:** Michael Vogeser, Monika Brüggemann, Kristina Brandt, Uta Ceglarek, Volker Gieskes, Niclas Hitziger, Andy Kahles, Ernst Klar, Dirk Roggenbuck, Henning Schliephake, Ortwin Schulte, Sascha Wettmarshausen, Uwe Zimmermann, Ulrich Sack, Albrecht Stenzinger, Arbeitsgemeinschaft der Wissenschaftlichen Medizinischen Fachgesellschaften e.V. (AWMF)

**Affiliations:** 1 Institut für Laboratoriumsmedizin, Klinikum der Universität München, LMU München, Deutschland; 2 Klinik für Innere Medizin II, Sektion für Hämatologische Spezialdiagnostik, Universitätsklinikum Schleswig-Holstein, Campus Kiel, Deutschland; 3 Zentrum für Klinische Studien, Universitätsklinikum Schleswig-Holstein, Campus Kiel, Deutschland; 4 Institut für Laboratoriumsmedizin, Klinische Chemie und Molekulare Diagnostik, Universitätsklinikum Leipzig, Deutschland; 5 Ministerium für Soziales, Gesundheit, Integration und Verbraucherschutz, Land Brandenburg, Potsdam, Deutschland; 6 Roche Diagnostics, Rotkreuz, Schweiz; 7 Institut für Pathologie, Universitätsklinikum Heidelberg, Deutschland; 8 AWMF Ad-hoc-Kommission Bewertung von Medizinprodukten, Berlin, Deutschland; 9 Medipan GmbH, Blankenfelde-Mahlow, Deutschland; 10 Klinik für Mund-, Kiefer- und Gesichtschirurgie, Georg-August-Universität Göttingen, Deutschland; 11 Bundesministerium für Gesundheit, Bonn, Deutschland; 12 Verband der Diagnostica-Industrie (VDGH e.V.), Berlin, Deutschland; 13 Deutsche Akkreditierungsstelle (DAkkS), Frankfurt, Deutschland; 14 Institut für Klinische Immunologie, Universitätsklinikum Leipzig, Deutschland; 15 Berlin, Deutschland

**Keywords:** IVDR

## Abstract

In order to discuss first experiences with the implementation of the EU Regulation on In Vitro Diagnostic Medical Devices (IVDR) about one year after its entry into force, the German Association of the Scientific Medical Societies (AWMF e.V.) organized a full-day public webinar. Overall, it became clear that the implementation of the IVDR still poses significant challenges for laboratory medicine and pathology. Corrections at the political level and implementation with a sense of proportion are required. Before the long-term goal of the IVDR, i.e. the increase in patient safety, can be realized, the prevention of disadvantages for patients due to gaps in care must be strived for in the medium term by all parties involved.

## Abstract (de)

Um etwa ein Jahr nach Inkrafttreten wesentlicher Teile der EU-Verordnung für In-vitro-Diagnostika (IVDR) erste Erfahrungen mit der Umsetzung zu diskutieren, wurde von der deutschen Arbeitsgemeinschaft der Wissenschaftlichen Medizinischen Fachgesellschaften (AWMF e.V.) ein ganztägiges, öffentliches Webinar veranstaltet. Insgesamt wurde dabei deutlich, dass die Implementierung der IVDR noch wesentliche Herausforderungen an die Labormedizin und Pathologie mit sich bringt. Korrekturen auf politischer Ebene und eine Umsetzung mit Augenmaß sind zu fordern. Bevor langfristig das Ziel der IVDR, nämlich die Erhöhung der Patientensicherheit, realisiert wird, ist mittelfristig vielmehr die Vermeidung von Nachteilen für Patientinnen und Patienten durch Versorgungslücken von allen beteiligten Kreisen anzustreben.

## Einleitung

Mit der EU-Verordnung für In-vitro-Diagnostika (IVDR) wird das Inverkehrbringen von In-vitro-Diagnostika (IVD) innerhalb der EU neu geregelt [1], [2], [3], [4], [5], [6], [7], [8], [9]. Die in der Arbeitsgemeinschaft der Wissenschaftlichen Medizinischen Fachgesellschaften (AWMF) organisierten Fachgesellschaften auf dem Gebiet der Laboratoriumsmedizin haben früh erkannt, dass sich aus der IVDR umfangreiche Konsequenzen für die Patientenbetreuung und für die Weiterentwicklung der Fachgebiete ergeben. Insbesondere bei der Bereitstellung innovativer, neuer Diagnostik, die oft als in-Haus-Test durchgeführt wird, bestand und besteht ein erheblicher Klärungs- und Unterstützungsbedarf sowohl auf Seiten der Labors als auch der Geschäftsführungen und Qualitätsmanager. Die AWMF hat daher im Juli 2019 die Ad-hoc-Kommission IVDR ins Leben gerufen, in der derzeit 18 wissenschaftliche Fachgesellschaften vertreten sind.

Als Verordnung ist die IVDR in den Mitgliedsstaaten direkt gültig; sie wurde im Mai 2017 vom EU-Gesetzgeber beschlossen und ist im Mai 2022 nach einer Übergangsphase in wesentlichen Teilen in Kraft getreten. 

In dieser Übergangsphase haben sich eine Reihe von Problembereichen der IVDR-Implementierung offenbart, die zu einer differenzierten Übergangsfristverlängerung einiger Vorgaben gezwungen hat. Das betraf vor allem die Unternehmen der IVD-Industrie, denen es zum Teil nicht möglich war, ihre Produkte fristgerecht nach den neuen Bedingungen bereitzustellen. Eine wesentliche Ursache hierfür war die mangelnde Verfügbarkeit von sogenannten Benannten Stellen, nicht-staatliche Unternehmen, die gemäß IVDR den Großteil der Diagnostika zu zertifizieren haben und für die die Europäische Kommission Detailvorgaben nur schleppend bereitstellte. 

Verglichen mit der Medical Device Regulation (MDR), die alle nicht-labordiagnostischen Medizinprodukte in neuer Weise adressiert, wird die IVDR in der Öffentlichkeit weniger wahrgenommen; gleichwohl stellt sie für die lückenlose und innovative Versorgung der Bevölkerung mit labordiagnostischen Leistungen eine erhebliche Herausforderung auf ganz unterschiedlichen Ebenen dar. Da die Marktverfügbarkeit von IVD für die Labordiagnostik fächerübergreifend von essenzieller Bedeutung ist, adressiert die Ad-hoc-Kommission In-vitro-Diagnostik der AWMF die IVDR-Implementierung intensiv. 

## Webinar der AWMF zur IVDR

Nach zwei vorhergehenden öffentlichen Symposien zu diesem Thema (Februar 2020 und Februar 2022; https://www.awmf.org/die-awmf/veranstaltungen/symposien) wurde am 17. März 2023 nun ein weiteres Webinar ausgerichtet, in dem insbesondere erste Erfahrungen möglichst vieler betroffener Kreise seit dem Inkrafttreten der IVDR erörtert werden sollten, um so ein Bild zum Stand der Umsetzung zu gewinnen. Die ganztätige Veranstaltung verzeichnete über 600 Teilnehmer, was die große Relevanz der IVDR für die diagnostischen Fächer verdeutlicht. In insgesamt 15 Vorträgen wurden Einschätzungen zum Implementierungsprozess der IVDR aus unterschiedlichen Perspektiven gegeben: Vertreten war das Bundesministerium für Gesundheit, die für die Überwachung des Medizinprodukterechtes zuständigen Behörden der Bundesländer, die Deutsche Akkreditierungsstelle, eine Benannte Stelle, Universitätsklinik-Labore, ein universitäres Studienzentrum sowie die Diagnostika-Industrie.

Herr **Ortwin Schulte** vom Bundesministerium für Gesundheit (BMG) berichtete, dass in der politischen Debatte zur IVDR auf europäischer Ebene eine Überkomplexität der Regelungsinhalte thematisiert wird. So wird im Europäischen Parlament deutliche Kritik am erreichten Umsetzungsstand der IVDR geäußert (u.a. im Hinblick auf die Funktionsfähigkeit des Register-Systems EUDAMED (Europäische Datenbank für Medizinprodukte) und der europäischen Referenzlabors für Hochrisikoprodukte) und Sympathie für die Verlagerung bestimmter Prozesse an die EMA (European Medicines Agency). Herr Schulte wies darauf hin, dass nach Art. 111 der IVDR für Mai 2027 eine Evaluation der IVDR vorgesehen ist, die in dringenden Fragen zu gesetzgeberischen Korrekturen führen kann. Das BMG misst im Hinblick auf diese Prozesse dem Dialog mit den Fachgesellschaften in der Labordiagnostik eine wichtige Bedeutung zu. 

Herr **Volker Gieskes**, Arbeitsgruppe Medizinprodukte der obersten Landesbehörden, wies darauf hin, dass sich auch die Rahmenbedingungen für die In-house-Herstellung von In-vitro-Diagnostika – die nicht in Verkehr gebracht werden – in erheblichem Ausmaß ändern. Die Einhaltung der grundlegenden Sicherheits- und Leistungsanforderungen gemäß Anhang I IVDR ist hierbei seit Mai 2022 verpflichtend. Andere Verpflichtungen, z.B. zur Aufrechterhaltung eines geeigneten Qualitätsmanagementsystems und zur Veröffentlichung von Informationen über In-house hergestellte IVDs, müssen im Mai 2024 umgesetzt sein. Erst im Mai 2028 tritt die Anforderung an eine Dokumentation in Kraft, aus der hervorgeht, dass die In-house hergestellten Produkte spezifische Anforderungen erfüllen, die von gleichartigen auf dem Markt befindlichen Produkten nicht erfüllt werden. Um die Umsetzung der Anforderungen aus der IVDR an die In-house-Herstellung zu erleichtern, wurde auf europäischer Ebene durch die Medical Device Coordination Group (MDCG) das Dokument „Guidance on the health institution exemption under Article 5(5)“ als Dokument „MDCG 2023-1“ veröffentlicht. In dem Dokument werden auf 17 Seiten und in 2 Anhängen mit umfangreichen Beispielen die Rahmenbedingungen für die In-house-Herstellung erläutert. Vor dem Hintergrund, dass auch die zuständigen Überwachungsbehörden dieses Dokument als Hilfestellung bei etwaigen Kontrollen nutzen, sollte dieses Dokument die Grundlage für die Überprüfung eigener Prozesse zur In-house-Herstellung sein.

**Professorin Uta Ceglarek**, Universitätsklinikum Leipzig, **Dr. Andy Kahles**, Universitätsklinikum Heidelberg, und **Professor Michael Vogeser**, LMU-Klinikum München, berichteten über die jeweiligen Ansätze und Verfahrensweisen im Hinblick auf eine angemessene und ressourcenbewusste Umsetzung der IVDR-Anforderungen im Hinblick auf die Eigenherstellung von IVD in universitären Laboren. Dabei wird ein recht weites Spektrum an Auslegungen einzelner Vorgaben deutlich. Dies betrifft u.a. das *produktspezifische* Risikomanagement, die produktspezifische Leistungsbewertung, die Spezifikation eines herstellungsspezifischen Qualitätsmanagementsystems sowie die Abgrenzung zwischen diagnostischen ärztlichen Leistungen (die Gegenstand der Selbstverwaltung des deutschen Gesundheitssystems sind) einerseits und der Herstellung von Artikeln, die auch durch die IVDR geregelt wird, andererseits. In dieser Hinsicht ist eine Schärfung der Begrifflichkeiten der IVDR zu fordern, um Überregulation zu vermeiden. Zu beachten ist dabei auch, dass nach Artikel 168 des EU-Vertrages die Regulation von Prozessen der medizinischen Versorgung dauerhaft in die Zuständigkeiten der Mitgliedsstaaten fällt. Es wurde auf eine Reihe von Rahmendokumenten hingewiesen, die die Ad-hoc-Kommission IVD der AWMF in den letzten Jahren erarbeitet und zweisprachig öffentlich zugänglich gemacht hat (https://www.awmf.org/service/arbeitshilfen-und-formulare#c1797). Erfahrungen zu möglichen behördlichen Überwachungen von Prozessen der Eigenherstellung existierten zum Tagungszeitpunkt noch nicht.

**Uwe Zimmermann**, Deutsche Akkreditierungsstelle (DAkkS), erörterte die Rolle der IVDR im Hinblick auf die Akkreditierung von medizinischen Laboratorien gemäß DIN EN ISO 15189. Dabei ist diese Norm die Grundlage der Kompetenzbestätigung; die Einhaltung von Anforderungen, die zusätzlich zu den Anforderungen der ISO 15189 festgelegt wurden, werden im Rahmen der Akkreditierung nicht explizit geprüft. Fällt Begutachtern jedoch ein Verstoß gegen gesetzliche Regelungen wie die IVDR auf, soll dies im Begutachtungsbericht vermerkt werden. 

**Kristina Brandt**, Zentrum für Klinische Studien, Universitätsklinikum Schleswig-Holstein, legte dar, dass die IVDR nicht nur im Hinblick auf eine Eigenherstellung von IVD für akademische Labore von Relevanz sein kann; auch für die Durchführung von Leistungsstudien für CE-zertifizierte IVD, die kommerzialisiert werden, sind wichtige regulatorische Anforderungen einzuhalten. 

**Dr. Sascha Wettmarshausen** hat Stand und Herausforderungen der IVDR-Implementierung aus Sicht des VDGH (Verband der Diagnostica-Industrie e.V.) als Branchenverband der deutschen Diagnostika-Industrie und von MedTechEuropa aus EU-Perspektive skizziert. Eine europaweite Umfrage unter den IVD-Herstellern, welche zwischen September und Oktober 2022 stattgefunden hat, zeigt den aktuellen Implementierungsstand der IVD-Verordnung auf. Unstrittig ist, dass die im Januar 2022 verabschiedete Änderungsverordnung (EU) 2022/112 eine erhebliche Erleichterung für das gesamte System gebracht hat. Die geänderten Übergangsbestimmungen erlauben risikobasiert die weitere Produktion und den Vertrieb von IVDs für Patienten, Labore und das gesamte Gesundheitssystem über Mai 2022 hinaus. 21 Prozent der gesamten Produktpalette sind zum Zeitpunkt der Veranstaltung bereits nach der Verordnung zertifiziert. Im Vergleich zum Vorjahr entspricht dies einer Verdreifachung. Gleichzeitig zeigt die Umfrage jedoch auch, dass es bis Mai 2025 noch zu Zertifizierungsengpässen kommen kann: 51 Prozent der IVDs der zukünftigen Hochrisikoprodukte der Klasse D gehören zu Herstellern, die noch keine Vereinbarung mit einer Benannten Stelle haben und somit noch nicht nach der IVDR zertifiziert werden können. Das betrifft mit 54 Prozent eine unverhältnismäßig große Anzahl kleiner und mittlerer Unternehmen (KMU). Ohne einen Vertrag mit einer Benannten Stelle können aber die allermeisten Produkte nicht nach der IVDR zertifiziert werden und stehen somit nach und nach dem Markt nicht mehr zur Verfügung. Herr Wettmarshausen unterstrich, dass es dringend notwendig ist, die Infrastruktur des Regulierungssystems rasch und einheitlich auszubauen, um drohenden Versorgungslücken zu begegnen. Vor allem die Konformitätsbewertungsprozesse, welche die Produkte zur Zertifizierung unter der IVDR bei Benannten Stellen durchlaufen, müssen kürzer, effizienter und vorhersehbarer werden. Ein solcher Prozess dauert derzeit im Schnitt zwischen 18 und 24 Monaten. Auch die nach der Verordnung bereits seit 2022 vorgesehene Etablierung von EU-Referenzlaboratorien für Hochrisikoprodukte ist noch in weiter Ferne. Derzeit sind Hersteller vor allem damit beschäftigt, ihre Bestandsprodukte zu überführen. Innovationen sind schwer bis gar nicht in den Markt zu bekommen. In der Konsequenz zeigt die Umfrage einen Rückgang von 28 Prozent bei den Herstellern, die der EU bei der ersten Produkteinführung den Vorzug geben würden.

Die weitreichenden Auswirkungen der IVDR-Implementierung in Großunternehmen der IVD-Industrie stellte Herr **Niclas Hitziger**, Roche Diagnostics, dar. Neben der erstmaligen Zertifizierung eines großen Teils der bestehenden Produkte ist mit der IVDR die „Post market surveillance and vigilance“ (PMSV) neu definiert worden und begleitet ein IVD-Produkt unter IVDR während des ganzen Produktlebenszyklus. Zu berücksichtigen ist auch, dass die IVDR-spezifischen Anpassungen auch in Ländern außerhalb der EU regulatorische Auswirkungen haben können, für die ebenfalls Zeit eingeplant werden sollte. Diese lokalen Registrierungsarbeiten in nicht-EU-Ländern muss berücksichtigt werden, damit die Umstellung des Produkts auf IVDR zum rechten Zeitpunkt erfolgt und so die globale Versorgung mit entsprechenden Produkten sichergestellt werden kann, was eine gut abgestimmte Logistik erfordert: Wenn die IVDR-spezifischen Anpassungen lokal noch nicht registriert sind, kann das auf IVDR umgestellte Produkt nicht in dieses Land importiert werden, was zu einem Versorgungsengpass führen kann. 

**Professor Dirk Roggenbuck** gab detaillierte Einblicke in die Dimensionen der IVDR-Implementierung aus der Perspektive eines mittleren Unternehmens. Die Implementierung der IVDR stellt für kleine und mittelständische Diagnostika Unternehmen in der europäischen Union hinsichtlich Innovationskraft und Wettbewerbsfähigkeit eine große Herausforderung dar. Diese ist gekennzeichnet durch signifikant erhöhte Kosten für regulatorische Aufwendungen vor allem in der neu umzusetzenden Überwachung in Verkehr gebrachter Produkte und die Einbeziehung einer Benannten Stelle in den Zertifizierungsprozess für die große Mehrzahl der diagnostischen Produkte inklusive Bestandsprodukte. Dies wird zur Verringerung der zertifizierten Produkte und einer eingeschränkten Innovationskraft führen. Vor allem für diagnostische Produkte mit relativ geringen Absatzzahlen, wie sie zum Beispiel für seltene Erkrankungen häufig anzutreffen sind, treffen diese Punkte zu. Die Geschäftsmodelle von kleinen und mittelständischen Unternehmen sind überdurchschnittlich in solchen Marktnischen angesiedelt. Obwohl die IVDR zur Erhöhung der Produktsicherheit durch das europäische Parlament verabschiedet wurde, gibt es keine gesamteuropäische Antwort, wie die damit verbundenen erhöhten Kosten über die in den einzelnen Mitgliedsstaaten unterschiedlichen Vergütungssystemen adressiert werden sollen. Da Änderungen der Vergütungsstruktur aufgrund der bestehenden Erfahrungen in Europa einen langen Zeitraum benötigen, werden Unternehmen mit begrenzten finanziellen Spielräumen sich großen Herausforderungen stellen müssen.

Die genannten Trends wurden am Beispiel der Medipan und GA Generic Assays GmbHs als verbundene brandenburgische Unternehmen mit 70 Mitarbeiter*innen dargestellt. Beide Unternehmen bieten zurzeit 150 diagnostische Testbestecke oder Gerätelösungen, welche unter der IVDD ohne Evaluierung über eine Benannten Stelle zertifiziert wurden, für die serologische Diagnose von autoimmunen Erkrankungen an. Mit der Transition zur IVDR wird sich die Anzahl der zertifizierten Produkte auf 86 verringern, wobei für 81 eine Einbeziehung einer Benannten Stelle notwendig wird. Die Kosten für die Evaluierung einer technischen Dokumentation werden auf 15.000 bis 45.000 € auf der Basis der aktuell vorliegenden Angebote der Benannten Stellen kalkuliert. Das bedeutet jährlich zusätzliche Kosten zwischen 60.000 und 240.000 € je nach Vorgaben für die Auswahl der zu evaluierenden technischen Dokumentationen durch die gewählte Benannte Stelle. Da die IVDR keine Kostenregulierung für Benannte Stellen, die als privatwirtschaftliche Unternehmen agieren, enthält und die Anzahl der Benannten Stellen in der europäischen Union auch in der nahen Zukunft begrenzt sein wird, ist mit keiner Kostenreduktion in diesem Bereich zu rechnen. 

Um die höheren regulatorischen Anforderungen zu adressieren, sind im Bereich Qualitätsmanagement und Regulatorische Angelegenheiten für beide Firmen insgesamt 6 hochqualifizierte Mitarbeiter mit geschätzten zusätzlichen jährlichen Kosten von 540.000 € notwendig. Aufgrund dieser zusätzlichen Kosten sind Preissteigerungen für die verbleibenden Produkte von 50 bis 100 Prozent notwendig, um das bestehende Geschäftsmodell für die Entwicklung und Vermarktung von diagnostischen Produkten für seltene Erkrankungen aufrecht zu erhalten. Beide Firmen planen in den nächsten 5 Jahren keine Einführung von neuen Produktentwicklungen. Es wurde die Suche nach und Evaluierung von neuen Geschäftsmodellen mit geringerem regulatorischen Aufwand, so zum Beispiel der Vermarktung von Reagenzien und Materialien für In-house-Diagnostika für Laboratorien, begonnen.

## Fazit und Position der AWMF

Insgesamt wurde in der ganztätigen Veranstaltung deutlich, dass die Implementierung der IVDR noch wesentliche Herausforderungen an die Labormedizin und Pathologie mit sich bringt. Die AWMF setzt sich nachdrücklich für notwendige Korrekturen auf politischer Ebene und eine Umsetzung der Verordnung mit Augenmaß ein. Bevor langfristig das Ziel der IVDR, nämlich die Erhöhung der Patientensicherheit, realisiert wird, ist mittelfristig vor allem die Vermeidung von Nachteilen für Patientinnen und Patienten durch Versorgungslücken von allen beteiligten Kreisen anzustreben. In der Bewertung der IVDR bis Mai 2027 entsprechend Artikel 111 muss nach Auffassung der AWMF-Ad-hoc-Kommission In-vitro-Diagnostik insbesondere deutlich darauf hingewiesen werden, dass eine – auf unscharfen Begrifflichkeiten beruhende – Anwendung der IVDR auf Prozesse und Verfahren im diagnostischen Labor zu überschießendem regulatorischen Aufwand führen würde, der die adäquate und innovative Patientenversorgung in den EU-Staaten gefährden würde. Eine entsprechende Präzisierung des Verordnungsinhaltes ist zu fordern.

## Anmerkung

Die Empfehlungen zur IVDR des Bundesverband Deutscher Pathologen e.V. (BDP) [7] sind für zahlende Mitglieder in drei Teilen auf der Website des BDP verfügbar:

- Teil 1: https://www.pathologie.de/?eID=downloadtool&uid=2233
- Teil 2: https://www.pathologie.de/?eID=downloadtool&uid=2234
- Teil 3: https://www.pathologie.de/?eID=downloadtool&uid=2239

## Interessenskonflikte

Die Autorinnen und Autoren erklären, dass sie keine Interessenkonflikte in Zusammenhang mit diesem Artikel haben. 
